# SARS-CoV-2 Infection Remodels the Phenotype and Promotes Angiogenesis of Primary Human Lung Endothelial Cells

**DOI:** 10.3390/microorganisms9071438

**Published:** 2021-07-03

**Authors:** Francesca Caccuri, Antonella Bugatti, Alberto Zani, Antonella De Palma, Dario Di Silvestre, Ekta Manocha, Federica Filippini, Serena Messali, Paola Chiodelli, Giovanni Campisi, Simona Fiorentini, Fabio Facchetti, Pierluigi Mauri, Arnaldo Caruso

**Affiliations:** 1 Section of Microbiology, Department of Molecular and Translational Medicine, University of Brescia Medical School, 25123 Brescia, Italy; antonella.bugatti@unibs.it (A.B.); a.zani033@unibs.it (A.Z.); e.manocha@unibs.it (E.M.); f.filippini020@unibs.it (F.F.); s.messali@unibs.it (S.M.); g.campisi@unibs.it (G.C.); simona.fiorentini@unibs.it (S.F.); 2 Proteomic and Metabolomic Laboratory, Institute of Biomedical Technologies, National Research Council (ITB-CNR), 20054 Segrate, Italy; antonella.depalma@itb.cnr.it (A.D.P.); dario.disilvestre@itb.cnr.it (D.D.S.); pierluigi.mauri@itb.cnr.it (P.M.); 3 Section of General Pathology, Department of Molecular and Translational Medicine, University of Brescia Medical School, 25123 Brescia, Italy; paola.chiodelli@unibs.it; 4 Pathology Unit, Department of Molecular and Translational Medicine, University of Brescia Medical School, 25123 Brescia, Italy; fabio.facchetti@unibs.it

**Keywords:** COVID-19, endothelial cell dysfunction, infection, angiogenesis, proteome

## Abstract

SARS-CoV-2-associated acute respiratory distress syndrome (ARDS) and acute lung injury are life-threatening manifestations of severe viral infection. The pathogenic mechanisms that lead to respiratory complications, such as endothelialitis, intussusceptive angiogenesis, and vascular leakage remain unclear. In this study, by using an immunofluorescence assay and in situ RNA-hybridization, we demonstrate the capability of SARS-CoV-2 to infect human primary lung microvascular endothelial cells (HL-mECs) in the absence of cytopathic effects and release of infectious particles. Preliminary data point to the role of integrins in SARS-CoV-2 entry into HL-mECs in the absence of detectable ACE2 expression. Following infection, HL-mECs were found to release a plethora of pro-inflammatory and pro-angiogenic molecules, as assessed by microarray analyses. This conditioned microenvironment stimulated HL-mECs to acquire an angiogenic phenotype. Proteome analysis confirmed a remodeling of SARS-CoV-2-infected HL-mECs to inflammatory and angiogenic responses and highlighted the expression of antiviral molecules as annexin A6 and MX1. These results support the hypothesis of a direct role of SARS-CoV-2-infected HL-mECs in sustaining vascular dysfunction during the early phases of infection. The construction of virus-host interactomes will be instrumental to identify potential therapeutic targets for COVID-19 aimed to inhibit HL-mEC-sustained inflammation and angiogenesis upon SARS-CoV-2 infection.

## 1. Introduction

Since December 2019, a new coronavirus belonging to Betacoronavirus named Severe Acute Respiratory Syndrome Coronavirus 2 (SARS-CoV-2) has become a global public health threat. The spectrum of clinical manifestation of SARS-CoV-2 infection includes mild to severe symptoms [1], whereas the prevalence of an asymptomatic form of this disease is proven [2,3]. In symptomatic patients, progression to pneumonia appears frequently. When SARS-CoV-2 infection progresses from mild to severe, patients may develop an acute respiratory distress syndrome (ARDS) followed by shock, tissue perfusion disorders, and multi-organ failure [1]. Classic ARDS reflects dramatic microvascular endothelial cell (mEC) dysfunction involving changes in vascular permeability, inflammation, activation of procoagulant pathways, and disruption of the alveolar-capillary barrier [4]. Evidence from several studies showed that coronavirus disease-19 (COVID-19) patients display these features. Pathological evaluation of lung autopsies has uncovered evidence of microvascular inflammation together with microvascular thrombi and the presence of viral particles associated with ECs [5,6]. Signs of vascular dysfunction included vascular endothelialitis and new vessel growth through a mechanism of intussusceptive angiogenesis [6]. These distinct angiocentric features of COVID-19, together with the evidence of SARS-CoV-2-infected ECs [5,6], indicate that viral-induced ARDS may be caused by a direct SARS-CoV-2 infection of human lung microvascular ECs (HL-mECs). However, this hypothesis is still debated since another study on post-mortem biopsies has shown that HL-mECs do not express SARS-CoV-2 antigens, thus suggesting that the observed endothelial damage in post-mortem biopsies may be due to an overwhelmed host inflammatory response rather than viral infection [7]. In the current perspective, the possibility to ascribe EC-related vascular dysfunction to a direct SARS-CoV-2 infection of HL-mECs is critical to understand SARS-CoV-2 pathogenic and therapeutic targets and demands stringent evidence-based studies.

A suitable in vitro cell model is essential for evaluating the capability of SARS-CoV-2 to infect HL-mECs and check the host response to the virus insult. MECs among different tissues are heterogeneous concerning their protein and surface marker expression [8,9], and such heterogeneity contributes to the diversity in their functions at different vascular sites [10,11], so in this study we have used primary HL-mECs for assessing their susceptibility to viral infection and their role in the vascular phase of COVID-19.

Here we show that HL-mECs sustain an abortive SARS-CoV-2 infection intracellularly since they express different viral proteins in the absence of infectious virus release. Infected HL-mECs were found to secrete pro-inflammatory cytokines and pro-angiogenic molecules. The SARS-CoV-2-conditioned microenvironment was also found to potently stimulate uninfected HL-mEC to acquire a pro-angiogenic phenotype. Intracellular profiles, performed by proteomic approach, demonstrated the remodeling of infected HL-mECs to antiviral, inflammatory, and angiogenic responses. All our data support the hypothesis that SARS-CoV-2-infected HL-mECs sustain inflammation and vascular dysfunction leading to vascular damage and leakage.

## 2. Materials and Methods

### 2.1. Cells

African green monkey kidney Vero E6 and Caco-2 cell lines were obtained from Istituto Zooprofilattico Sperimentale della Lombardia e dell’Emilia Romagna (Brescia, Italy) and maintained in Dulbecco’s Modified Eagle Medium (DMEM; Gibco, Thermo Fisher Scientific, Waltham, MA, USA) supplemented with 10% fetal bovine serum (FBS; Gibco, Thermo Fisher Scientific). Human breast cancer cells (MDA-MB 231) were obtained from the American Type Culture Collection (ATCC, Manassas, VA, USA) and grown in DMEM supplemented with 10% FBS. HL-mECs were purchased from Lonza Clonetics (Walkersville, MD, USA) and cultured in EGM-2 MV (Lonza, Basel, Switzerland) containing 10% FBS. Human Aortic Endothelial Cells (HAEC) were isolated in our laboratory [12] and cultured in EGM-2 (Lonza) containing 10% FBS.

### 2.2. Viral Infection

Infection experiments were run using the clinical SARS-CoV-2 isolate AP66 as previously described [13], which maps the Wuhan reference strain and carries the D614G mutation. The virus was propagated in Vero E6 cells and the viral titer was determined by a standard plaque assay. All the experiments were performed with a single viral inoculum. The inactivation of aliquots of viral stock was achieved by UV treatment, according to Patterson et al. [14]. To check viral inactivation, UV-treated SARS-CoV-2 was used to infect Vero E6 cells. The absence of viral replication was assessed by monitoring the lack of cytopathic effects and viral antigens’ expression by immunofluorescence. Mock-infected cell cultures were obtained from uninfected cells, processed exactly as the SARS-CoV-2-infected ones. We performed infection experiments in a biosafety level-3 (BLS-3) laboratory at an MOI of 0.05 or 1. When reported, HL-mECs were pre-treated for 30 min at 37 °C with 30 µg/mL of GRGDSPK (RGD) or GRADSPK (RAD) peptides (Neosystem Laboratoire, Strasbourg, France) before SARS-CoV-2 infection.

### 2.3. Viral RNA Extraction and qRT-PCR

RNA was extracted from clarified cell culture supernatants (16,000 g × 10 min) and infected cells using QIAamp Viral RNA^®^ Mini Kit (Qiagen, Hilden, Germany) and RNeasy Plus mini kit (Qiagen), respectively, according to the manufacturer’s instructions. RNA was eluted in 30 μL of RNase-free water and stored at −80 °C until use. The qRT-PCR was carried out following previously described procedures [15]. Briefly, reverse transcription and amplification of the spike (S) gene were performed using the one-step QuantiFast Sybr Green RT-PCR mix (Qiagen) as follows: 50 °C for 10 min; 95 °C for 5 min; 95 °C for 10 s; 60 °C for 30 s (40 cycles) (primers: RBD-qF1: 5′-CAA TGG TTT AAC AGG CAC AGG-3′ and RBD-qR1: 5′-CTC AAG TGT CTG TGG ATC ACG-3). A standard curve was obtained by cloning the receptor-binding domain of the S gene (primers: RBD-F: 5′-GCT GGA TCC CCT AAT ATT ACA AAC TTG TGC C-3′; RBD-R: 5′-TGC CTC GAG CTC AAG TGT CTG TGG ATCAC-3′) into pGEM T-easy vector (Promega, Madison, WI, USA). A standard curve was generated by the determination of copy numbers derived from serial dilutions (10^3^–10^9^ copies) of the plasmid. Each quantification was run in triplicates. The 2^−ΔΔCq^ (Livak) method was used for the comparison of the target qPCR product with the standard curve.

### 2.4. Metagenomic Analysis

RNA was eluted in 30 μL and stored at −80 °C until use. Randomly amplified cDNA was generated using the Sequence-Independent Single-Primer Amplification (SISPA) Round A/B technique as previously described [13]. To maximize the recovery of fragments > 200 bp, PCR products were purified using 1.8 × ratio AMPure XP beads (Agencourt, Beckman Coulter Inc., Pasadena, CA, USA). Purified products were quantified using the Qubit^®^ DNA HS Assay Kit (Thermo Fisher Scientific, Waltham, MA, USA), then genomic libraries were prepared using Nextera DNA Flex kit (Illumina, San Diego, CA, USA). Sequencing was performed using an Illumina MiniSeq^®^ platform (Illumina) generating 2 × 150 bp paired-end reads, then were trimmed with Trimmomatic ver. 0.3880 for quality (Q score > 25) and length (>36 bp), as previously described [16]. Raw data were also checked for quality using FastQC (https://www.bioinformatics.babraham.ac.uk/projects/fastqc/, accessed on 16 July 2020) and for bacterial, archaeal, and viral genomes correspondence using Kraken2 with MiniKraken2 Database [17]. Paired-end trimmed reads were analyzed with Geneious^®^ software (version 11.1.5) (Biomatters Ltd., Auckland, New Zealand). The consensus sequence was reconstructed and mapped to the SARS-CoV-2 reference sequence NC_045512.2 using Bowtie2 in sensitive-local mode with a consensus threshold of 65% [18]. Datasets generated have been deposited in the Genbank repository (accession numbers ERR4691983: SARS-CoV-2-UNIBS-AP66-P1V-0320; ERR4659294: SARS-CoV-2-UNIBS-AP66-HMVEC-0320).

### 2.5. RNA In Situ Hybridization

Cultured cells were fixed in 10% neutral formaldehyde solution for 24 h, centrifuged for 10 min at 3000 rpm, and washed by centrifugation in physiologic solution for 10 + 10 min at 3000 rpm. Plasma and HemosIL8 RecombiPlasTin 2G (Instrumentation Laboratory, Bedford, MA, USA) were added to the pellet dropwise until the formation of a clotted sphere which was embedded in paraffin. Next, 2 µm thick sections were used for in situ hybridization applying the V-nCov2019-S-antisense probes specific for the S gene encoding the spike protein (accession NC_045512.2; Advanced Cell Diagnostics, Hayward, CA, USA), using RNAscope 2.5 HD Detection Reagent-Red (Advanced Cell Diagnostic) in accordance with the manufacturer’s protocol, adopting an extended 1 h incubation in Amp 5 and 30 min incubation in Amp 6. All samples were counterstained with Harris’s hematoxylin. Digital photographs were obtained using the Olympus DP73 digital camera and the cellSens Dimension software (Olympus Corporation, Milan, Italy).

### 2.6. Immunofluorescence Assay

Uninfected cells were seeded (5 × 10^4^ cells per well) in 8-well chamber slides (Becton–Dickinson, Franklin Lakes, NJ, USA) and then infected as described above. After infection, cells were fixed with 2% paraformaldehyde in phosphate-buffered saline (PBS) for 10 min, permeabilized with 0.1% Triton X100 in PBS, and saturated with 3% bovine serum albumin (BSA), 0.1% Tween 20 in PBS. For staining, cells were incubated overnight with a human serum containing IgG to SARS-CoV-2 (1:1000 dilution), with an anti-SARS-CoV-2 spike (S) glycoprotein monoclonal antibody (1:1000 dilution; abcam, clone 1A9, Cambridge, UK), anti-SARS-CoV-2 nucleocapsid (NP) monoclonal antibody (1:1000 dilution; abcam, clone 6H3), anti-SARS-CoV-2 envelope (E) polyclonal antibody (1:1000 dilution; abcam) followed by Alexa Fluor 488-conjugated anti-human, mouse or rabbit IgG (Thermo Fisher Scientific). Nuclei were counterstained with 4′,6-diamidino,2-phenylindole (DAPI, Merck, Darmstadt, Germany). Cells were photographed under a Zeiss Axiovert 200 M epifluorescence microscope equipped with a Plan-Apochromat 40x or 63x/1.4 NA oil objective. Z-stack images acquired using the ApoTome imaging system were elaborated through the AxioVision Extended Focus module (Zeiss Axiovert 200 M system).

### 2.7. Tube Formation Assay

Tube formation assays were performed as previously described, with minor modifications [19]. Briefly, 150 µL of Cultrex Reduced Growth Factor Basement Membrane Extract (RGF BME) (Trevigen Inc., Gaithersburg, MD, USA) were transferred to prechilled 48-well culture plates. Plates were incubated for 1 h at 37 °C. Cells were resuspended in the culture medium containing 10% FBS, seeded 4.5 × 10^4^ per well, and analyzed for the tube formation at 12 h after cell seeding by examination with a Leica DM IRB microscope. The center of each well was digitally photographed with a Hitachi KP-D50 camera and capillary-like structures were quantified by analyzing the number of tubes per well, formed by ECs. Fibroblast growth factor 2 (FGF-2; Santa Cruz Biotechnology, Dallas, TX, USA) at 100 ng/mL was used as the positive control.

### 2.8. Spheroids Assay

Spheroids were generated by mixing HL-mECs (2 × 10^5^ cells/mL) with 5 mg/mL of methylcellulose (Sigma-Aldrich Inc., St. Louis, MO, USA) in EGM-2 MV medium containing 10% FBS, making the final volume 10 mL. The cells (100 µL/well) were then added to 96-well plates (Greiner Bio-one, Kremsmünster, Austria) and incubated at 37 °C, 5% CO_2_ for 24 h. FGF-2 (Santa Cruz Biotechnology) at 100 ng/mL was used as the positive control.

Separately, the collagen I gel solution (Rat tail, Corning Incorporated, Corning, NY, USA) was maintained on ice and neutralized by adding NaOH 0.1 M and PBS 10X to a final pH of 7.4. Then, the 24-well plates were coated with neutralized collagen (200 µL/well) and incubated in a humidified 5% CO_2_ incubator for 1 h at 37 °C. The spheroids from 96-well plates were collected in Eppendorf tubes and centrifuged at 4000 rpm for 10 s. When a clear pellet was distinguished, the supernatant was removed, and the pellet was kept in a volume of about 100 µL collagen I-neutralized solution. Each collagen-spheroid mixture was rapidly added to the precoated 24-well plates (100 µL/well) and incubated for 1 h. After 1 h, 500 µL of EGM-2 MV containing 10% FBS, was added to the wells to cover the surface completely and plates were further incubated for 24 h. Sprouting occurred from the spheroid core, photographed with a Hitachi KP-D50 camera, and the number of sprouts was counted with the spheroids of similar sizes from three different wells of the plate.

### 2.9. Co-Cultivation

Co-culture between infected and not infected HL-mECs was performed in the absence of direct cell contact by using transwell inserts (polycarbonate filters, coated with collagen, 0.4 µm pore size, Corning, New York, NJ, USA). Briefly, HL-mECs seeded in the lower compartment were infected as described above, while not infected HL-mECs were seeded on the collagen-coated inserts in the upper chamber. After 3 days, cells in the upper well were trypsinized and used to perform the tube formation or spheroid assay.

### 2.10. Microarray Analysis

Supernatants from infected HL-mECs were collected at different time points (1, 2, and 3 days post-infection (p.i.)), clarified, and analyzed for the expression of 55 different angiogenesis-related proteins by Human Angiogenesis Array Kit (Proteome Profiler, R&D systems, Minneapolis, MN, USA) or for the expression of 36 different cytokine-related proteins by the Human Cytokine Array Kit (Proteome Profiler, R&D systems) according to the manufacturer’s instructions.

### 2.11. Protein Extraction and Enzymatic Digestion

The proteomic analysis described in this study was performed on mock-, SARS-CoV-2-, and UV-SARS-CoV-2-infected HL-mECs, collected at days 1 and 3 p.i. For each condition, about 2 × 10^6^ cells were used and two preparations were examined. Cells were lysed and proteins were extracted, reduced/alkylated, and enzymatically digested using Easy PepTM Mini MS Sample Prep Kit (Thermo Fisher Scientific, Rockford, IL, USA). Tryptic and Lys-C digestion was carried out on 100 µg and 70 µg of the two examined preparations, respectively. Following the kit protocol, in less than 3 h and for each examined condition, peptides were generated, cleaned-up to prepare detergent-free samples, and resuspended in 0.1% formic acid (Sigma-Aldrich Inc., St. Louis, MO, USA) for liquid chromatography-tandem mass spectrometry (LC-MS/MS) analysis.

### 2.12. LC-MS/MS Analysis

Peptide mixtures were analyzed using the Eksigent nanoLC-Ultra^®^ 2D System (AB SCIEX Dublin, CA, USA) configured in trap-elute mode. Briefly, samples (0.8 µg injected) were first loaded on a trap (200 × 500 µm ChromXP C18-CL, 3 µm, 120 Å) and washed with the loading pump running in isocratic mode with 0.1% formic acid in water for 10 min at a flow of 3 µL/minute. The automatic switching of the autosampler ten-port valve then eluted the trapped mixture on a nano reversed-phase column (75 µm × 15 cm ChromXP C18-CL, 3 µm, 120 Å) through a 133 min gradient of eluent B (eluent A, 0.1% formic acid in water; eluent B, 0.1% formic acid in acetonitrile) at a flow rate of 300 nL/minute. In-depth, the gradient was: from 5–10% B in 3 min, 10–40% B in 110 min, 40–95% B in 12 min, and holding at 95% B for 8 min. The eluted peptides were directly analyzed on an LTQ-OrbitrapXL mass spectrometer (Thermo Fisher Scientific) equipped with a nanospray ion source. The spray capillary voltage was set at 1.7 kV and the ion transfer capillary temperature was held at 220 °C. Full MS spectra were recorded over a 400–1600 m/z range in positive ion mode, with a resolving power of 60,000 (full width at half-maximum) and a scan rate of 2 spectra/second. This step was followed by five low-resolution MS/MS events that were sequentially generated in a data-dependent manner on the top five ions selected from the full MS spectrum (at 35% collision energy), using the dynamic exclusion of 0.5 min for MS/MS analysis. Mass spectrometer scan functions and high-performance liquid chromatography solvent gradients were controlled by the Xcalibur data system version 1.4 (Thermo Fisher Scientific).

### 2.13. Data Handling

All data generated were searched using the Sequest HT search engine contained in the Proteome Discoverer software, version 2.1 (Thermo Fisher Scientific, Waltham, CA, USA). Experimental MS/MS spectra were compared with the theoretical mass spectra obtained by in silico digestion of 29 SARS-CoV-2 protein sequences obtained from the Uniprot (www.uniprot.org) and Homo Sapiens proteome database (74,842 entries), downloaded from Uniprot in January 2020. The following criteria were used for the identification of peptide sequences and related proteins: trypsin and Lys-C as enzymes; methionine oxidation; carbamidomethyl at cysteines; three missed cleavages per peptide; mass tolerances of ±50 ppm for precursor ions; and ±0.8 Da for fragment ions. Percolator node was used with a target-decoy strategy to give a final false discovery rate (FDR) at a Peptide Spectrum Match (PSM) level of 0.01 (strict) based on q-values, considering a maximum deltaCN of 0.05 [20]. Only peptides with a minimum peptide length of six amino acids, and rank 1 were considered. Protein grouping and strict parsimony principles were applied. Results were then exported to an Excel file for further processing.

### 2.14. Differential Expression, Linear Discriminant Analysis, and PPI Network

The 45 protein lists (mock-infected HL-mECs: Day 1, n = 5, Day 2, n = 5, Day 3, n = 5; SARS-CoV-2-infected HL-mECs: Day 1, n = 5, Day 2, n = 5, Day 3, n = 5; UV-SARS-CoV-2-infected HL-mECs: Day 1, n = 5, Day 2, n = 5, Day 3, n = 5), obtained from the SEQUEST algorithm, were aligned, normalized, and compared utilizing the average peptide spectrum matches (aPSM) [21], corresponding to the average of all the spectra identified for a protein and, consequently, to its relative abundance, in each analyzed condition (mock-, SARS-CoV-2- and UV-SARS-CoV-2-infected HL-mECs). In-depth, to select differentially expressed proteins, mock-, SARS-CoV-2-, and UV-SARS-CoV-2-infected HL-mECs were pairwise compared, applying a threshold of 0.3 and 5 on the two MAProMa indexes DAve (Differential Average) and DCI (Differential Confidence Index), respectively. In addition, the protein lists were processed by linear discriminant analysis (LDA) and proteins with the largest F ratio and smallest *p*-value (< 0.05) were retained. Proteins selected by LDA, DAve, and DCI were processed by hierarchical clustering applying Ward’s method and the Euclidean distance metric using JMP 15.2 software.

A protein–protein interaction (PPI) network (212 nodes and 1785 edges) was built by combining differentially expressed proteins (n = 214) and the Homo sapiens PPI network retrieved from the STRING database [22]; only experimentally and database defined PPIs with a score > 0.15 were considered. The resulting sub-network was visualized and analyzed by Cytoscape and its plugins [23]. Proteins were grouped in functional modules by the support of BINGO87 and STRING; about BINGO, Homo sapiens organism, hypergeometric test, and Benjamini–Hochberg FDR correction, a significance level 0.01 was applied whereas the default setting was used for STRING.

### 2.15. qRT-PCR

Total RNA was extracted from Caco-2, MDA-MB-231, and HL-mEC cells using the RNeasy Plus Mini Kit (Qiagen, Hilden, Germany) and reverse transcribed (Applied Biosystems, Foster City, CA, USA). Quantitative Real-Time PCR was performed on the 7500 Real-Time PCR system (Applied Biosystems) with ACE2 (Hs01085333_m1) and ACTB (beta-actin; Hs01060665_g1) TaqMan gene expression assays (Thermo Fisher Scientific).

### 2.16. Western Blot Analysis

Protein samples (30 µg) obtained from lysis in RIPA buffer (Cell Signaling Technology, Danvers, MA, USA) of Caco-2, MDA-MB-231, and HL-mEC cells were separated on 10% sodium dodecyl sulfate-polyacrylamide gel electrophoresis and then transferred onto polyvinylidene difluoride membranes (Millipore, Sigma, Burlington, MA, USA). After being blocked with 3% BSA in tris buffered saline buffer containing 0.05% Tween 20, the blot was probed with mouse anti-human ACE2 monoclonal antibody (1:500 dilution; Santa Cruz Biotechnology; clone E-11) and with mouse anti-human GAPDH monoclonal antibody (1:1000 dilution; Santa Cruz Biotechnology; clone G-9). The antigen–antibody complexes were detected using peroxidase-conjugated goat anti-mouse IgG (Sigma) and revealed using the enhanced chemiluminescence (ECL) system (Santa Cruz Biotechnology).

### 2.17. Statistical Analysis

Data were analyzed for statistical significance using the Student’s two-tailed *t*-test or one-way ANOVA when appropriate. The Bonferroni post test was used to compare data. Differences were considered significant when *p* < 0.05. Statistical tests were performed using Prism 8 software (GraphPad Software, La Jolla, CA, USA).

## 3. Results

### 3.1. Abortive Infection of SARS-CoV-2 in HL-mECs

**Figure 1:**
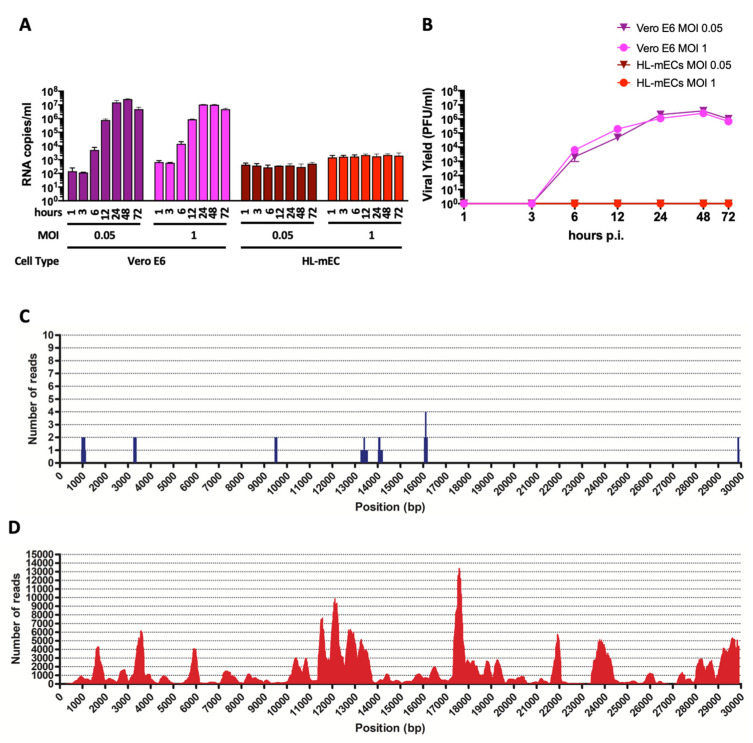
SARS-CoV-2 does not productively infect HL-mECs. (**A**) Vero E6 and HL-mECs were infected with SARS-CoV-2 at a MOI of 0.05 or 1. The graph shows SARS-CoV-2 genome quantitation in cell supernatants collected at 1, 3, 6, 12, 24, 48, and 72 h p.i. by qRT-PCR. Values represent the S gene copies/mL mean ± SD of a triplicate. (**B**) Supernatants were collected from Vero E6 and HL-mECs at 1, 3, 6, 12, 24, 48, and 72 h p.i. and infectious viral titers were determined by plaque assay. Data represent the average of three replicates from one experiment. (**C**) Deep sequencing analysis of SARS-CoV-2 viral progeny released from HL-mECs or (**D**) from Vero E6 cells at day 3 p.i. Whole-genome sequences of SARS-CoV-2 released in the supernatants were performed by metagenomic analyses. Obtained reads were mapped on the virus genome reference NC_045512.2 (*x*-axis) for genome reconstruction; the y-axis shows the number of reads mapped at each nucleotide position.

Human primary lung microvascular endothelial cells (HL-mECs) and Vero E6 cells were infected for 1 h at 37 °C with the SARS-CoV-2 isolate AP66 (available at GenBank, ERR4961983: SARS-CoV-2-UNIBS-AP66: P1V-0320) at either low (0.05) or high (1) multiplicity of infection (MOI). SARS-CoV-2 caused, as expected, a strong cytolytic effect on the Vero E6 cell monolayer at both MOIs at day 3 p.i. (Supplementary Figure S1A). At the same time, SARS-CoV-2 did not show any cytopathic effect on HL-mECs at days 3 and 7 p.i. (Supplementary Figure S1B). Moreover, SARS-CoV-2-infected HL-mECs cells were largely viable for up to 7 days p.i. and could be regularly split. Quantification of RNA released by Vero E6 cells over time shows that viral production is very similar at the MOI of 0.05 and 1 (Figure 1A). A very low amount of virus-specific RNAs was present in the supernatant of SARS-CoV-2-infected HL-mECs at 1 h p.i. and was considered the basal value of the experiment. SARS-CoV-2 RNA levels did not show any increase over time, suggesting that HL-mECs do not support active virus replication (Figure 1A). A plaque assay confirmed the absence of infectious virions in the supernatant of HL-mECs (Figure 1B). To finally prove that HL-mECs do not support virus release, cellular supernatant from infected cells at the highest MOI of 1 were harvested at 3 days p.i. and analyzed for the presence of virus-specific sequences by metagenomic Illumina sequencing. As shown in Figure 1C, only 17 out of the total 4,508,784 trimmed reads (available at GenBank, ERR4659294: SARS-CoV-2-UNIBS-AP66-HMVEC-0320) mapped on the reference SARS-CoV-2 sequence NC_045512.2 as well as on the AP66-SARS-CoV-2 isolate. A further evaluation of the raw reads excluded the presence of any defective/recombined SARS-CoV-2 sequence within the analyzed dataset. As expected, Vero E6 cells infected in parallel to HL-mECs with the same viral inoculum released an abundant amount of infectious SARS-CoV-2 (Figure 1C,D). As high as 403,530 out of 3,638,840 total trimmed reads were identified as SARS-CoV-2 specific reads.

**Figure 2:**
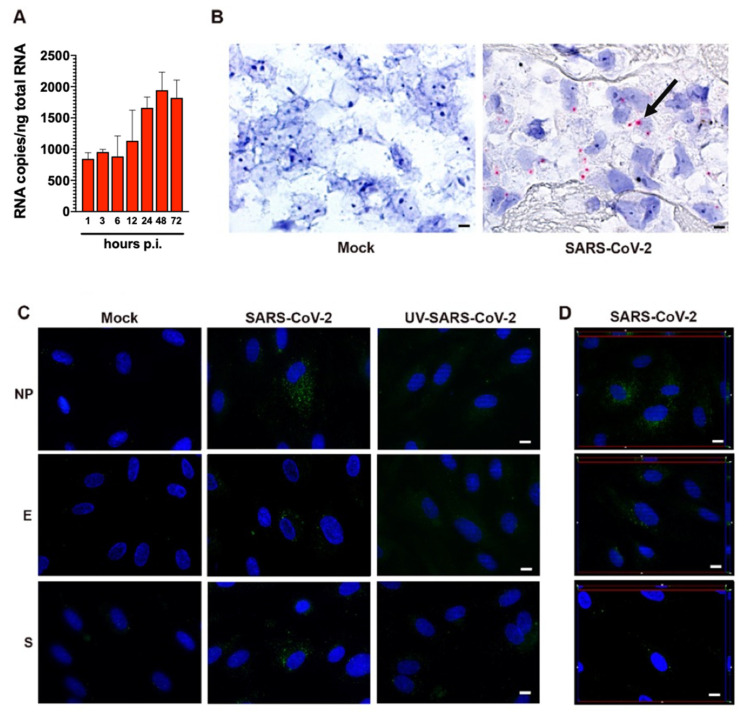
SARS-CoV-2-infected HL-mECs express viral RNA and proteins. (**A**) Quantitation of SARS-CoV-2 genomes at the intracellular level by qRT-PCR. At least three replicates were performed. Data are representative of two independent experiments with similar results. (**B**) SARS-CoV-2 RNA in situ hybridization using the S antisense probe shows strong expression of viral RNA as red cytoplasmic dots in SARS-CoV-2-infected HL-mECs (black arrow), while mock-infected cells (Mock) are negative (scale bar, 3 µm). (**C**) HL-mECs were mock-infected (Mock) or infected with SARS-CoV-2 (SARS-CoV-2) or with UV-inactivated SARS-CoV-2 (UV-SARS-CoV-2) at MOI 1, for 1 h at 37 °C, then washed and cultured until day 3 p.i. Immunofluorescence was performed by incubating cells with antibodies targeting the SARS-CoV-2 NP (upper panels), E (middle panels), or S (lower panels) proteins (scale bar, 10 µm). (**D**) z-Stack sections and orthogonal z reconstitution of SARS-CoV-2-infected HL-mECs expressing NP, E, or S proteins (scale bar, 10 µm). Images of HL-mECs display SARS-CoV-2 signals in green and cell nuclei in blue.

As shown in Figure 2A, quantification of intracellular SARS-CoV-2 RNA in HL-mECs showed the presence of a low number of viral RNA copies. No significant increase in intracellular viral RNA copies was observed over time, suggesting a lack of progression in viral genome synthesis during the time. At the same time, SARS-CoV-2 RNA in situ hybridization, using the S antisense probe, revealed intense signal positivity in the form of cytoplasmic dots in about 12% of HL-mECs, while mock-infected cells were negative (Figure 2B).

Due to the presence of viral RNA in HL-mECs, we asked whether it could be translated into proteins. HL-mECs were infected with SARS-CoV-2 at an MOI of 1 and tested for the intracellular expression of viral proteins by immunofluorescence assay. In order to exclude a carry-over of viral proteins from the initial inoculum, we tested HL-mECs infected with 1 MOI of a UV-inactivated SARS-CoV-2 (UV-SARS-CoV-2) inoculum [14] (Supplementary Figure S1C) for viral protein expression. As shown in Supplementary Figure S2, viral proteins were detected in HL-mECs at days 1, 2, and 3 p.i. by using a human serum containing IgG to SARS-CoV-2 as a specific reagent, whereas no viral protein expression was observed in mock-infected and in UV-SARS-CoV-2-infected HL-mECs. This finding confirms that the replicating virus does undergo active RNA translation into proteins in HL-mECs. Approximately 10% of SARS-CoV-2-infected HL-mECs were found to express spike (S), nucleocapsid (NP), and envelope (E) proteins by using specific monoclonal and polyclonal antibodies, whereas mock- and UV-SARS-CoV-2-infected cells resulted as negative (Figure 2C). Taken together, these results suggest that HL-mECs may support the expression of virus-specific transcripts but are devoid of a fully permissive phenotype.

### 3.2. SARS-CoV-2-Infected HL-mECs Release Inflammatory Cytokines and Angiogenic Molecules

**Figure 3:**
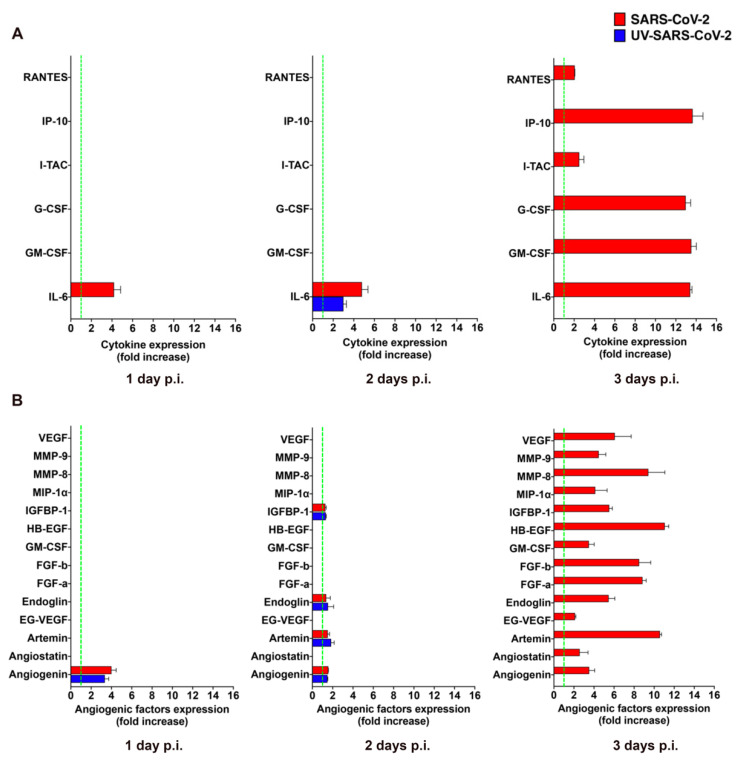
SARS-CoV-2 promotes the release of cytokines and angiogenic molecules from HL-mECs. HL-mECs were treated with infectious (SARS-CoV-2) or UV inactivated (UV-SARS-CoV-2) SARS-CoV-2 at MOI 1, for 1 h at 37 °C and then washed and cultured until day 3 p.i. Supernatants of SARS-CoV-2 and UV-SARS-CoV-2 HL-mECs were evaluated at 1 (left panel), 2 (middle panel), and 3 (right panel) days p.i. for the presence of (**A**) cytokines or (**B**) angiogenic molecules by human proteome arrays. The results are expressed as mean values ± SD of duplicates given as fold increase as compared to mock-infected cells. Data are representative of one out of three independent experiments with similar results.

The clinical progression of COVID-19 to critical illness is associated with an exaggerated immune response, leading to the magnified inflammation termed “cytokine storm”, which is thought to contribute to the pathogenicity of COVID-19 progression [24]. Virus-infected mECs are known to release different pro-inflammatory cytokines and pro-angiogenic molecules upon viral infection [12,25,26] that promote mEC injury and contribute to the development of microcirculatory lesions and thrombosis [27]. To understand whether the abortive infection of SARS-CoV-2 was impacting the biological function of HL-mECs, we performed an analysis of the secretome at days 1, 2, and 3 p.i., by using a human cytokine array. As shown in Supplementary Figure S3A, mock-infected HL-mECs did not spontaneously release any of the tested cytokines but little amounts of interleukin (IL)-6 at day 2 and 3 of culture only. As shown in Figure 3A, SARS-CoV-2 was not able to induce the secretion of any pro-inflammatory cytokine at days 1 and 2 p.i. over mock-infected cells, with the only exception of IL-6, that resulted in a 4.0 ± 0.6 and 4.6 ± 0.6 fold-increase at day 1 and 2 p.i., respectively. However, SARS-CoV-2 triggered the secretion of interferon gamma-induced protein (IP)-10, granulocyte colony-stimulating factor (G-CSF), granulocyte-macrophage colony-stimulating factor (GM-CSF), and IL-6, powerfully, together with small amounts of regulated on activation, normal T cell expressed and secreted (RANTES) and interferon-inducible T cell alpha chemoattractant (I-TAC) at day 3 p.i., compared to mock-infected cells. Release of cytokines over the control levels was not observed in the supernatants collected at day 1, 2, and 3 p.i. from HL-mECs infected with the UV-SARS-CoV-2, with the only exception of IL-6 that showed a 3.0 ± 0.6-fold increase over the control at day 2 p.i. Our results demonstrate that SARS-CoV-2 promotes the secretion of different pro-inflammatory cytokines that takes place almost exclusively on day 3 p.i.

GM-CSF is a potent pro-angiogenic factor [28], able to induce human EC proliferation [29], as well as maturation and stabilization of new microvessels [30]. This finding prompted us to investigate the capability of SARS-CoV-2 to promote the secretion of other pro-angiogenic factors. As shown in Supplementary Figure S3B, analysis of the mock-infected HL-mEC secretome did not show the spontaneous release of any of the tested angiogenic factors during the 3 days of observation, except for a transient expression of heparin binding-epidermal growth factor (HB-EGF) and GM-CSF observed at day 2 of culture. Angiogenin was the only observed angiogenic molecule transiently increased in the secretome of SARS-CoV-2- and UV-SARS-CoV-2-infected HL-mECs (3.9 ± 0.4 and a 3.4 ± 0.4-fold-increase over mock-infected cells, respectively) at day 2 p.i. On the other hand, a plethora of pro-angiogenic factors was detected in the secretome obtained at day 3 p.i. from SARS-CoV-2–but not from UV-SARS-CoV-2–infected HL-mECs (Figure 3B). The angiogenesis array confirmed the increased secretion of GM-CSF by viral infected ECs compared to control cells, together with other potent angiogenic molecules as fibroblast growth factor (FGF)-a and -b, HB-EGF, matrix metalloproteinases (MMP)-8 and 9, insulin growth factor binding protein (IGFBP)-1, angiogenin, and endoglin. Of interest was the huge expression of Artemin in the secretome of infected cells, since this is the first evidence on the capability of human ECs to produce and secrete this potent pro-angiogenic factor upon viral infection. This finding is in keeping with the up-modulation of both vascular endothelial growth factor (VEGF) and endocrine gland-derived vascular endothelial growth factor (EG-VEGF) found in the secretome of SARS-CoV-2-infected HL-mECs since Artemin acts via VEGF expression and signaling [31].

### 3.3. SARS-CoV-2 Triggers EC Angiogenic Functions

**Figure 4:**
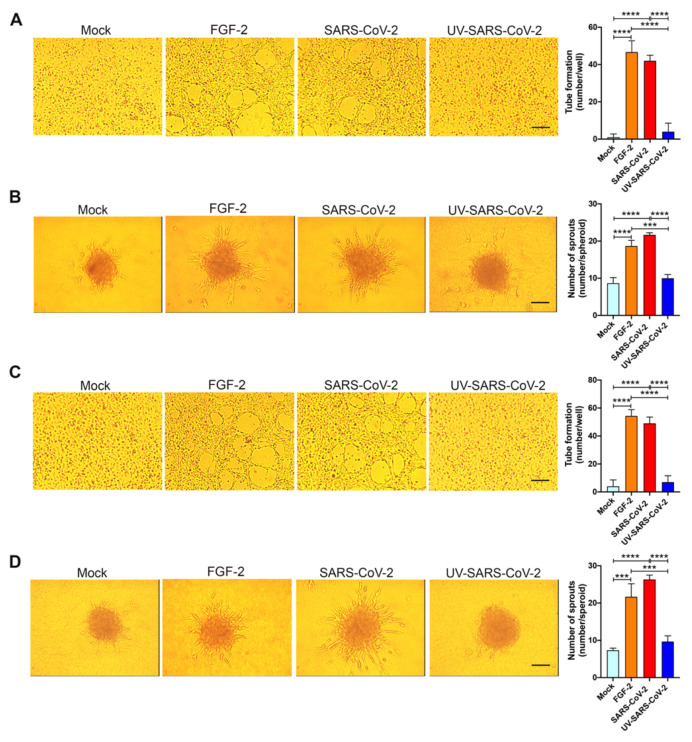
SARS-CoV-2 induces angiogenesis in HL-mECs by conditioning the microenvironment. HL-mECs were mock-infected (Mock) or infected with SARS-CoV-2 (SARS-CoV-2) or UV-inactivated SARS-CoV-2 (UV-SARS-CoV-2) at MOI 1, for 1 h at 37 °C and then washed and cultured until day 3 p.i. (**A**) Mock-, SARS-CoV-2-, and UV-SARS-CoV-2 HL-mECs were seeded on reduced growth factor Matrigel-coated wells and then cultured for 12 h at 37 °C. Pictures are representative of one out of three independent experiments with similar results (scale bar, 200 µm). (**B**) Sprouting of spheroids generated with Mock, SARS-CoV-2, and UV-SARS-CoV-2 HL-mECs. Pictures are representative of one out of three independent experiments with similar results (scale bar, 10 µm). (**C**) Tube formation assay performed with HL-mECs co-cultivated for 3 days with Mock, SARS-CoV-2, or UV-SARS-CoV-2 HL-mECs. The pictures were taken 12 h after cell seeding (scale bar, 200 µm). Pictures are representative of one out of three independent experiments with similar results. (**D**) Sprouting of spheroids generated with HL-mECs co-cultivated for 3 days with Mock, SARS-CoV-2, or UV-SARS-CoV-2 HL-mECs. Pictures are representative of one out of three independent experiments with similar results (scale bar, 10 µm). Values reported are the mean ± SD of one representative experiment out of three independent experiments with similar results performed in triplicate. Statistical analysis was performed by one-way ANOVA and Bonferroni’s post test was used to compare data (*** *p* < 0.001, **** *p* < 0.0001).

To explore the angiogenic function of SARS-CoV-2-infected HL-mECs, we examined their capacity to form tube-like structures. On day 3 p.i., cells were seeded on 48-well plates (4.5 × 10^4^ per well) containing polymerized plugs of growth factor-reduced BME. As shown in Figure 4A, mock-infected HL-mECs seeded on growth factor-reduced BME and cultured for 12 h formed a cellular monolayer. At the same time, SARS-CoV-2-infected HL-mECs developed a consistent network of tube-like structures. This finding attests to a potent, self-sustaining, viral-induced angiogenic activity. Spontaneous angiogenesis was not observed in UV-SARS-CoV-2-infected HL-mECs, thus demonstrating that the presence of viral antigens at the intracellular level is needed to prime HL-mECs toward a strong angiogenic phenotype.

The entrapment of EC spheroids in biopolymeric gels represents a three-dimensional (3D) cell model that was found to be an attractive potential method of forming a microvascular network that mimics in vivo sprouting angiogenesis. The 3D organotypic culture is based on the property of HL-mECs to form spheroids under nonadherent conditions, and it has proven useful for studies on endothelial capillary sprouting [32]. Modifying the recently established lymph node-derived lymphoid EC spheroid differentiation model [19], we developed a spheroidal system of HL-mEC aimed at mimicking the correct 3D assembly of the lung capillary blood vessel wall. HL-mECs infected with SARS-CoV-2, UV-SARS-CoV-2, or mock-infected were collected at day 3 p.i. and spheroids were prepared according to our previously described method [19]. As shown in Figure 4B, control, mock-infected, as well as UV-SARS-CoV-2-infected spheroids, did not show any sprout formation after 24 h of observation, while at the same time, a dramatic outgrowth of sprouts was observed in SARS-CoV-2-infected spheroids. These data strongly support the role of SARS-CoV-2 in triggering HL-mEC angiogenesis.

To understand whether the angiogenic activity promoted by SARS-CoV-2 could be extended to other ECs, we performed key angiogenic experiments on primary HAEC as a model of macrovascular ECs. As shown in Supplementary Figure S4, mock-infected HAECs seeded on growth factor-reduced BME and cultured for 12 h formed a cellular monolayer. On the contrary, SARS-CoV-2-infected HAECs were able to form a consistent network of tube-like structures thus attesting to the ability of SARS-CoV-2 to trigger macrovascular ECs’ angiogenic activity.

### 3.4. SARS-CoV-2 Promotes a Pro-Angiogenic Microenvironment

To assess whether angiogenesis was due to a direct effect of virus antigen expression within HL-mECs or had to be ascribed to the conditioned microenvironment promoted by infected cells, we investigated the effect of the SARS-CoV-2-infected HL-mEC secretome on its not-infected counterpart, using a co-culture assay. As shown in Figure 4C, HL-mECs co-cultured for 72 h in the collagen-coated upper insert well of a 0.4 μm pore-size Transwell with SARS-CoV-2-infected HL-mECs in the lower chamber, acquired the ability to form a consistent network of tube-like structures when detached and seeded for 12 h on the growth factor-reduced BME. On the contrary, HL-mECs co-cultured with mock-infected or UV-SARS-CoV-2-infected HL-mECs were unable to exert spontaneous angiogenesis. Similar results were observed using the spheroid assay. As shown in Figure 4D, sprouts were observed in HL-mECs spheroids generated from cells co-cultured with SARS-CoV-2-infected HL-mECs only. Our data highlight that the SARS-CoV-2-conditioned microenvironment is directly responsible for the angiogenic features acquired by not infected HL-mECs.

The angiogenic activity observed in HL-mECs upon SARS-CoV-2 infection was almost superimposable and not statistically different when compared to that induced by the SARS-CoV-2-conditioned microenvironment on uninfected HL-mECs.

### 3.5. Proteome Analysis of SARS-CoV-2-Infected HL-mECs

**Figure 5:**
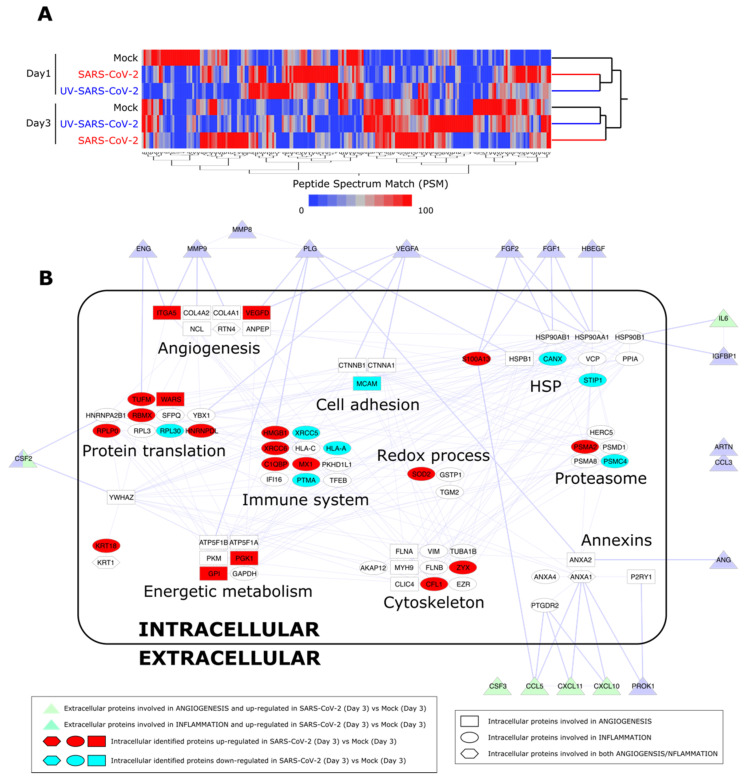
Proteome analysis of SARS-CoV-2-infected HL-mECs. (**A**) Hierarchical clustering of HL-mEC proteomes at day 1 and 3 p.i. The dendrogram was obtained by computing the average peptide spectrum matches (aPSM) of the 214 DEPs selected by LDA and MAProMa software; the Euclidean distance metric and Ward’s method were applied (JMP15.2 software) with DAve index > 0.3. (**B**) PPI network at day 3 p.i. of DEPs involved in angiogenesis and inflammation. The interactome network (91 nodes and 262 edges) was built through the mapping of intracellular proteins found differentially expressed by proteomics and extracellular proteins assayed by angiogenic and inflammation array of SARS-CoV-2 vs. Mock HL-mECs. Physical or/and functional interactions connecting intra- and extra-cellular identified proteins are highlighted by thicker edges. Intracellular node color code indicates proteins up-regulated (in red) and down-regulated (in blue light) in SARS-CoV-2- vs. mock-infected HL-mECs. For more details see Materials and Methods Section 2.14.

In order to confirm a remodeling of HL-mECs to inflammatory and angiogenic responses following SARS-CoV-2 infection, cell content was studied by a label-free approach based on nano-liquid chromatography coupled to high-resolution mass spectrometry (nLC-hrMS). We identified a total of 1974 proteins in the host proteomes (Supplementary Table S1). Combining LDA and MAProMa software, 214 differentially expressed proteins (DEPs) were extracted (Supplementary Table S2). DEPs were then processed by hierarchical clustering. As shown in Figure 5A, a different behavior over time of the three analyzed conditions (mock, SARS-CoV-2, and UV-SARS-CoV-2) was demonstrated. In particular, at day 3 p.i., SARS-CoV-2-infected HL-mECs resulted as distinct from UV-SARS-CoV-2-infected and mock-infected cells, which grouped in the same branch. Focusing on the biological processes triggered in the SARS-CoV-2 secretome at day 3 p.i., the most significant DEPs detected in HL-mECs, related to angiogenesis and inflammation, were considered for further analyses. A PPI network was built, and the selected proteins were represented as nodes grouped into functional sub-modules. Figure 5B shows the resulting PPI network and highlights the relationship among intracellular proteins and the secreted ones, assayed with the angiogenic and inflammation arrays. The same analysis of UV-SARS-CoV-2-infected versus mock-infected HL-mECs is reported in Supplementary Figure S5. Supplementary Table S3 reports the up- and down-regulated DEPs at day 1 to 3 in infected cells, using both mock and UV-infected cells as controls. The most affected submodules referring to the translation and homeostasis of proteins, energetic metabolism, and immune system, are in agreement with the recent findings reported for SARS-CoV-2-infected Caco-2 [33,34] and Vero E6 [35] cells. Of note, viral proteins were not detected at any day p.i., attesting for a limited translation of the SARS-CoV-2 genome in HL-mECs and/or to the low number of cells permissive to viral infection. Nevertheless, some cellular proteins related to virus infection resulted as significantly increased at day 1 p.i. and remodeled at day 3 p.i. Among them, it is worth highlighting peptidyl prolyl isomerase A (PPIA), also knowns as cyclophilin A, increases in a broad range of inflammatory diseases, including viral infections. PPIA is a natural ligand for basigin (BSG), also known as CD147. The BSG/PPIA pathway was found better represented in HL-mECs than in the lung and airway epithelial cells [36], and was shown to sustain inflammation in a number of diseases, including acute lung inflammation, while displaying also potent immune cell chemotactic properties [37,38]. More recently, a role for the BSG/PPIA axis in SARS-CoV-2 entry into different ECs was proposed [39].

A similar behavior in SARS-CoV-2-infected HL-mECs was observed for annexins (ANXs): ANXA1 and ANXA2, which enhance viral replication, and mainly ANXA6, which is known to strongly contrast Influenza A virus replication [40]. As shown in Supplementary Table S2, and Supplementary Figures S6 and S7, ANXA6 strongly increased at day 1 p.i. in SARS-CoV-2-infected HL-mECs compared to mock-infected cells, whereas it decreased at day 3 p.i., suggesting its possible role in controlling SARS-CoV-2 replication. This decline was concomitant to an increased expression of the interferon-inducible protein MX1 at day 3 p.i., which is known to contrast a wide range of RNA viruses, including Influenza A [41,42]. Other interferon-related proteins, such as WARS1, GAPDH, HERC5, IFI16, VCP, and VIM were identified (see Supplementary Table S2).

Other molecules that increased at day 3 p.i. in SARS-CoV-2-infected HL-mECs deserve attention for their role in maintaining HL-mEC homeostasis: proteasome 20S subunit alpha 2 (PSMA2) and proteasome 26S subunit, ATPase 4 (PSMC4), both components of the 26S proteasome which catalyzes protein degradation, and Zyxin (Zyx), a protein implicated in actin cytoskeleton remodeling, but also displaying a crucial role in thrombin signaling in ECs. Indeed, Zyx has been reported to activate the protease-activated receptor 1 (PAR-1) signaling pathway and sustain thrombosis [43]. As expected, different proteins involved in angiogenesis were found upregulated in SARS-CoV-2-infected HL-mECs at day 3 p.i., compared to control cells (Supplementary Figure S7), confirming the relevance of this biological process in the investigated phenotypes. In particular: tryptophan-tRNA ligase (WARS 1), one of the proteins of the translation module known to regulate the extracellular-signal-regulated kinases (ERK), protein kinase B (Akt), and endothelial nitric oxide synthase (eNOS) activation pathways associated with angiogenesis and is secreted in response to interferon [44]; VEGF-D, involved in the activation of VEGF receptors on the EC surface, thus contributing to promoting EC growth, migration [45], and also blood vessel permeability and pulmonary edema in acute lung injury under hyperoxic conditions [46]; alpha V integrin (ITGA5), which is one of the major endothelial fibronectin receptors, playing a key role in cell proliferation, tissue repair, inflammation, infection, and angiogenesis [47]; glucose-6-phosphate isomerase (G6PI or GPI), known to promote angiogenesis under hypoxia conditions [48].

### 3.6. Integrin-Dependent SARS-CoV-2 Entry into HL-mECs

**Figure 6:**
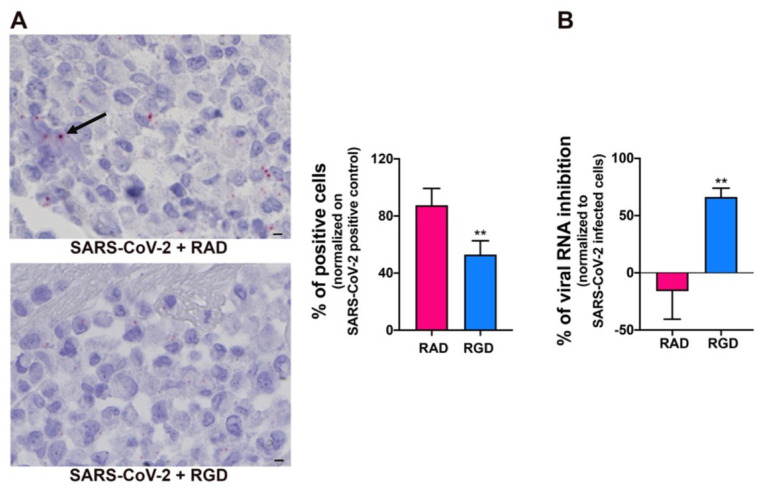
Integrins partially mediate SARS-CoV-2 infection of HL-mECs. HL-mECs were treated or not with RAD or RGD peptides before SARS-CoV-2 infection. (**A**) SARS-CoV-2 RNA in situ hybridization (black arrow) using the S antisense probe (scale bar, 3 µm). (**B**) Quantitation of SARS-CoV-2 genomes at the intracellular level by qRT-PCR. At least three replicates were performed. Data are representative of two independent experiments with similar results. Statistical analysis was performed by the Student’s two-tailed *t*-test (** *p* < 0.01).

SARS-CoV-2 utilizes the host angiotensin-converting enzyme 2 (ACE2) for binding and entry into the host cells [49]. In a normal adult human lung, ACE2 is expressed primarily in alveolar epithelial type II cells, which are supposed to be the main target and, possibly, a reservoir of the virus [50]. ACE2 expression on different organ-derived mECs [49,51,52], including HL-mECs both in vitro and in vivo is still debated [53,54]. Since our proteome analysis did not show ACE2 expression in HL-mECs, we asked whether this finding was due to the lack of sensitivity of the assay. ACE2 expression was then evaluated on HL-mECs by real-time PCR and Western blotting. As shown in Supplementary Figure S8, ACE2 expression was not detected at both mRNA and protein levels. Recently, it has been demonstrated that the SARS-CoV-2 spike protein interacts with integrins expressed on the cell surface through its conserved RGD motif (403–405: Arg-Gly-Asp) [55]. Our proteomic analysis showed that different integrins, ITGA5 in particular, are expressed in HL-mECs. Therefore, we determined the involvement of integrins in SARS-CoV-2 entry into ECs by using the peptide RGD, an inhibitor of integrin–ligand interactions. SARS-CoV-2-infected-HL-mECs treated with the control peptide RAD showed a strong positivity for SARS-CoV-2, almost superimposable to control, untreated, and infected cells, whereas the signal was significantly reduced in the RGD peptide-treated cells (Figure 6A). This finding was further confirmed by a significant reduction in intracellular viral RNA level in RGD-peptide-treated compared to RAD-peptide-treated cells (Figure 6B). These data support a role for integrins in mediating SARS-CoV-2 entry into HL-mECs.

## 4. Discussion

To date, the capability of SARS-CoV-2 to infect mECs is controversial since transmission electron microscopy analysis of lung biopsies showed the presence of SARS-CoV-2 into lung ECs [6], while other investigators disagreed on image interpretation [13,56,57]. Here, we show that HL-mECs sustain an abortive SARS-CoV-2 replication since we observed the intracellular expression of viral RNA and proteins in the absence of infectious viral progeny release. This occurred in the absence of ACE2 expression, which is considered indispensable for active SARS-CoV-2 replication in ECs [54]. Although a low-level expression of ACE2—or limited to a cell subset according to the known EC heterogeneicity [58]—cannot be completely ruled out, our data suggest the capability of SARS-CoV-2 to use an alternate receptor to infect HL-mECs. Many viruses have an arginine-glycine-aspartic acid (RGD) tripeptide motif displayed on their viral envelope glycoproteins, which is the minimal peptide sequence for binding integrins [59]. As an integrin recognition motif, RGD plays an important role in virus infection as a means of cell surface binding and virus internalization [59]. Recently, it has been demonstrated that SARS-CoV-2 may interact with integrins through the conserved RGD motif at position 403–405 of its spike protein [55]. It is worth noting that integrins capable of interacting with the RGD motif include ITGA5 [59], whose expression was highlighted in SARS-CoV-2-infected-HL-mECs by our proteomic analysis. Based this evidence, we performed experiments aimed to assess the role of integrins in SARS-CoV-2 infection of HL-mECs. We show for the first time that SARS-CoV-2 utilizes integrins for entry into HL-mECs. It is worth noting that ACE2 overexpression on HL-mECs results in high viral titers, multi-nucleate syncytia, and cell lysis [54], thus attesting for the full capacity of HL-mECs to sustain lytic viral replication. On the contrary, SARS-CoV-2 entry into ACE2 negative HL-mECs results in abortive infection. This finding may be explained by the lack of critical virus/host interactions needed for a complete viral life cycle, and/or, by activation of a potent innate antiviral response. This latter hypothesis is corroborated by our proteomic analysis, which shows an innate response of HL-mECs to the virus insult. In particular, an increased expression of ANXA6 is observed soon after HL-mEC infection, which declines over time concomitantly to the enhanced expression of MX1. Both molecules are known to strongly contrast a wide range of RNA viruses, including Influenza A [40,41,42]. In particular, MX1 is highlighted as a critical responder in SARS-CoV-2 infection with its expression levels being higher in COVID-19 than in non-COVID-19 patients and strikingly correlated to viral load increment [60]. Moreover, in SARS-CoV-2-infected macaques, a significant up-regulation of MX1 was shown by transcriptomic analysis and immunohistochemistry, confirming viral sensing at the lung level [61]. The ability of MX1 to localize to coat protein I (COPI)-positive membranes of the smooth endoplasmic reticulum/Golgi intermediate compartment [62] merits attention since it has been hypothesized to be essential for inhibiting viruses that rely on membranes to replicate, as positive-strand RNA viruses including SARS-CoV [63].

Expression of SARS-CoV-2 antigens triggered an altered secretory pattern leading to a pro-inflammatory and pro-angiogenic microenvironment. This finding supports the hypothesis that infected ECs may directly contribute to immune activation, vascular permeability, and endothelialitis [5,61]. ARDS and acute lung injury are life-threatening manifestations of COVID-19. As for classic ARDS, SARS-CoV-2-associated ARDS reflects a critical HL-mEC dysfunction involving changes in inflammation, vasculogenesis, activation of procoagulant pathways, and vascular permeability leading to diffuse damage of the alveolar-capillary barrier [4,6]. Histologic evidence of lung endothelial barrier degradation is concomitant to an intense CD4^+^ and CD8^+^ T cell infiltrate [64], suggesting a role of immune-mediated injury in the pathophysiology of severe COVID-19. Excessive infiltration of activated alveolar inflammatory macrophages, neutrophils, and platelets into alveoli and lung interstitial tissue is suggested to further contribute to inflammation and vascular damage [65]. These histological pieces of evidence are resembling another acute lung inflammatory process such as malaria-associated ARDS [66]. Understanding the pathogenic mechanisms that drive immune dysfunction and vascular leakage is crucial for the development of effective treatments.

Many inflammatory cytokines have been reported to be elevated in COVID-19 patients and be a determinant of pathological alterations and clinical manifestations of ARDS [67]. Patients with severe COVID-19 exhibited higher levels of pro-inflammatory cytokines than patients with mild or moderate symptoms [68,69]. Recent data point to the key role of activated, highly inflammatory (M1 polarized) alveolar macrophages in sustaining a cytokine storm [24] and promoting lung vascular EC activation and consequently, vascular permeability, leakage, and immune cell extravasation [70]. Differently, our data highlight a primary direct role of HL-mECs in promoting the release of several biologically active molecules following SARS-CoV-2 infection. From this point of view, HL-mECs act as innate immune cells, able to produce and secrete high levels of pro-inflammatory molecules such as IL-6, but also chemokines recruiting T cells, monocytes, and neutrophils into the inflammatory site, such as IP-10 [71], I-TAC [72], RANTES [73], and growth factors promoting immune cell activation and maturation, such as G-CSF and GM-CSF [74,75].

Expression of viral RNA and proteins inside HL-mECs was found to be necessary to generate also a potent pro-angiogenic microenvironment, capable of inducing trans-differentiation of not-infected HL-mECs into elements performing spontaneous angiogenesis. Angiogenesis is a perfectly balanced effect between pro- and anti-angiogenic molecules, with an angiogenic switch “on” when the net balance is tipped in favor of angiogenesis [76]. An imbalance of angiogenic regulators is responsible for aberrant angiogenesis, with neo-formed vessels being structurally and functionally abnormal, displaying widened inter-endothelial junctions, a discontinuous or absent basement membrane, and leakage [77]. Indeed, HL-mECs in the specimens from patients with COVID-19 show disruption of intercellular junctions, cell swelling, and a loss of contact with the basal membrane [6]. Therefore, the aberrant intussusceptive angiogenesis described in lung biopsies obtained from COVID-19 patients [6] may be likely referred to as a pro-angiogenic microenvironment directly promoted by SARS-CoV-2-infected HL-mECs.

Another possible mechanism at work for sustaining pulmonary vascular leakage in severe COVID-19 patients can be ascribed to the role of mECs as conditional innate immune cells [78]. MECs participate in inflammation via interactions with specialized effector cells and by acting as antigen-presenting cells (APCs) [79]. Although mECs are not professional APCs, their role in antigen presentation has been recognized [80,81]. This makes mECs capable of actively participating in both innate and adaptive immunity. Indeed, activated mECs effectively induce transendothelial migration of antigen-specific CD4^+^ and CD8^+^ T cells and trigger them to produce cytokines and proliferate [82]. Moreover, previous studies showed that mECs are also capable of antigen cross-presentation to CD8^+^ T cells [83,84]. It is worth noting that HL-mEC antigen cross-presentation to CD8^+^ T cells was found to be the main mechanism driving malaria-associated ARDS and acute lung injury [66]. Moreover, dexamethasone has been shown to be the only drug able to decrease CD8^+^ T cells infiltration in the lungs and protect malaria-infected mice from lung pathology [85]. It is worth noting that dexamethasone decreases mortality in patients with severe COVID-19 [86]. Defining whether SARS-CoV-2 abortive infection may activate HL-mECs to act as APCs and stimulate the activity of immune cells in promoting and/or sustaining a pro-inflammatory and pro-angiogenic microenvironment, deserves further studies. It is worth noting that the capability of SARS-CoV-2 to induce an abortive infection is not only limited to HL-mECs but it has already been postulated in other cell targets [87].

In conclusion, the concept of COVID-19 as an endothelial disease, at least with respect to its pulmonary complications, has been already envisaged [88]. Our data point to a unifying pathophysiological picture of SARS-CoV-2-associated ARDS and lead us to hypothesize a key role of SARS-CoV-2-infected HL-mECs in sustaining inflammation, aberrant angiogenesis, and chemoattraction of immune cells in the early phases of viral infection. Our hypothesis is strengthened by the recent finding that inflammatory pathways occur in bronchoalveolar lavage of macaques as early as day 1 following the SARS-CoV-2 challenge, whereas macrophage infiltrates occurred on days 2 and 4 p.i. in large airways, thus filling alveoli throughout regions of consolidation and abnormal pathology [61]. Knowledge of the viral antigen(s) driving HL-mEC dysregulation and the construction of virus–host interactomes will be instrumental to develop antiviral agents aimed to disrupt virus–host protein–protein interactions.

## Data Availability

Datasets generated have been deposited in the Genbank repository (accession numbers ERR4691983: SARS-CoV-2-UNIBS-AP66-P1V-0320; ERR4659294: SARS-CoV-2-UNIBS-AP66-HMVEC-0320).

## Supplementary Materials

The following are available online at https://www.mdpi.com/article/10.3390/microorganisms9071438/s1, Figure S1–S8, Tables S1–S3. Figure S1. SARS-CoV-2 infection does not induce any cytopathic effect on HL-mECs. Figure S2. SARS-CoV-2-infected HL-mECs express viral proteins. Figure S3. Release of cytokines and angiogenic molecules from mock-infected HL-mECs. Figure S4. SARS-CoV-2 induces angiogenesis in HAECs. Figure S5. PPI network of DEPs involved in angiogenesis and inflammation at day 3 p.i. in UV-SARS-CoV-2 vs. Mock-Infected HL-mECs. Figure S6. PPI network of DEPs involved in angiogenesis and inflammation at day 1 p.i. Figure S7. Differential analysis (by Dave) of the main extracted proteins. Figure S8. ACE2 expression in HL-mECs. Table S1. Identified proteins in the host proteomes. Table S2. Differentially expressed proteins. Table S3. Up- and down-regulated differentially expressed proteins at day 1 to 3 in infected cells.
