# Supplementary material for: SARS-CoV-2 Infection Remodels the Phenotype and Promotes Angiogenesis of Primary Human Lung Endothelial Cells
Source: Microorganisms. 2021 Jul 3;9(7):1438. doi: 10.3390/microorganisms9071438 (PMC8305478; doi:10.3390/microorganisms9071438)
Supplement: Supplementary file 1 [file microorganisms-09-01438-s001.zip › Supplementary Figures.pdf]

Supplementary Figures

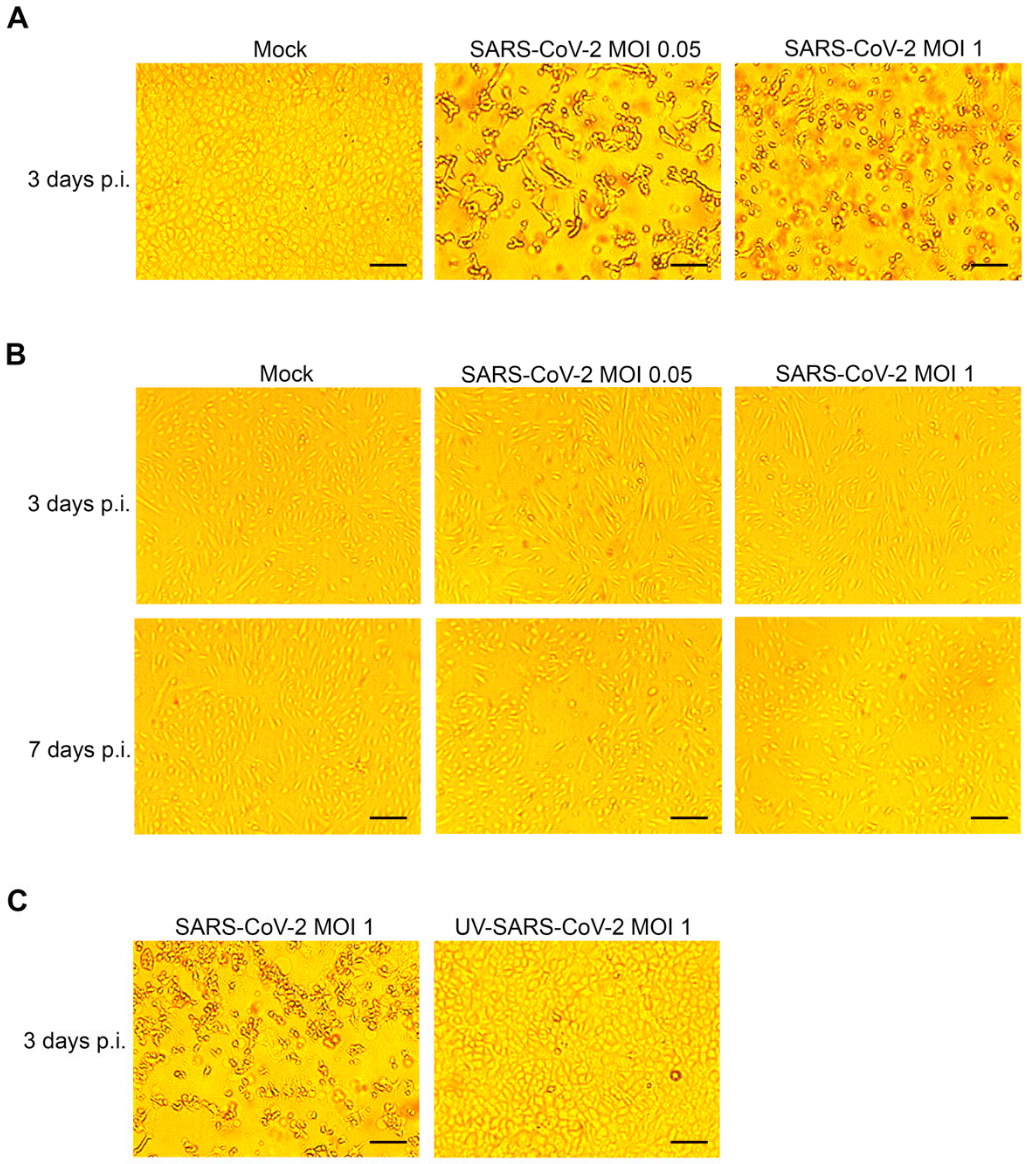

Figure S1. SARS-CoV-2 infection does not induce any cytopathic effect on HL-mECs. (**A**) Vero E6 were mock-infected (Mock) or infected with SARS-CoV-2 (SARS-CoV-2) at a MOI of 0.05 or 1, for 1 hour at 37°C and then washed and cultured in fresh medium. Images show light microscopy evaluation of Mock (left panel), and SARS-CoV-2 at MOI 0.05 or 1 at day 3 p.i. (Scale Bar, 100 µm). (**B**) HL-mECs were infected as above and cultured for 3 and 7 days p.i.. Images show light microscopy evaluation of Mock and SARS-CoV-2 at MOI 0.05 or 1 at day 3 and 7 p.i. (Scale Bar, 100 µm). (**C**) Vero E6 cells were infected with SARS-CoV-2 (SARS-CoV-2) or with UV-inactivated SARS-CoV-2 (UV-SARS-CoV-2) at a MOI of 1, for 1 hour at 37°C and then washed and cultured in fresh medium. Light microscopy images show the evaluation of SARS-CoV-2 and UV-SARS-CoV-2 at day 3 p.i. (Scale Bar, 100 µm).

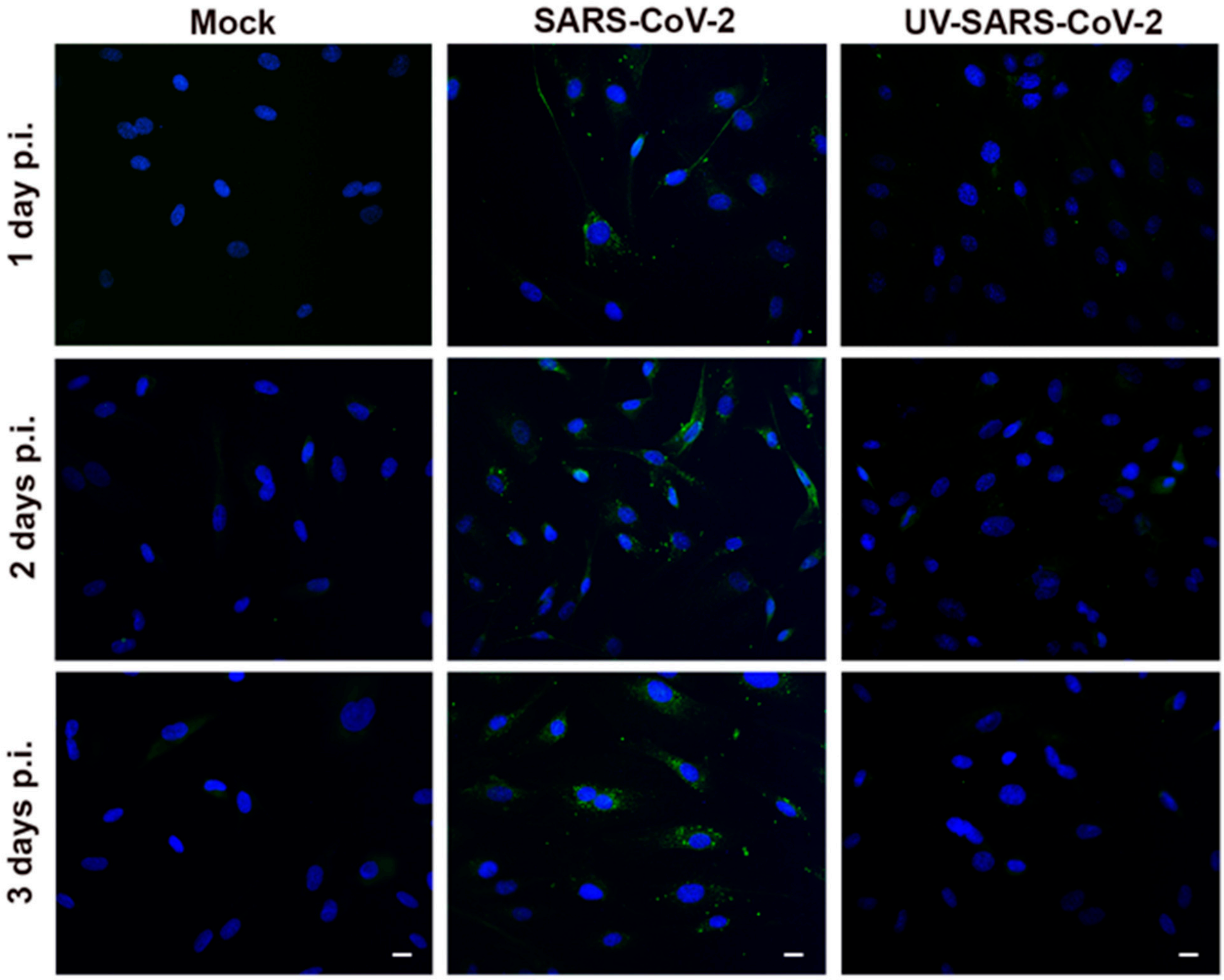

Figure S2. SARS-CoV-2-infected HL-mECs express viral proteins. HL-mECs were mock-infected (Mock) or infected with SARS-CoV-2 (SARS-CoV-2) or with UV-inactivated SARS-CoV-2 (UV-SARS-CoV-2) at MOI 1, for 1 hour at 37°C, then washed and cultured until day 3 p.i.. Immunofluorescence was performed by incubating cells with a human serum containing IgG to SARS-CoV-2 (Scale Bar, 10 µm). Images of HL-mECs display SARS-CoV-2 signals in green and cell nuclei in blue.

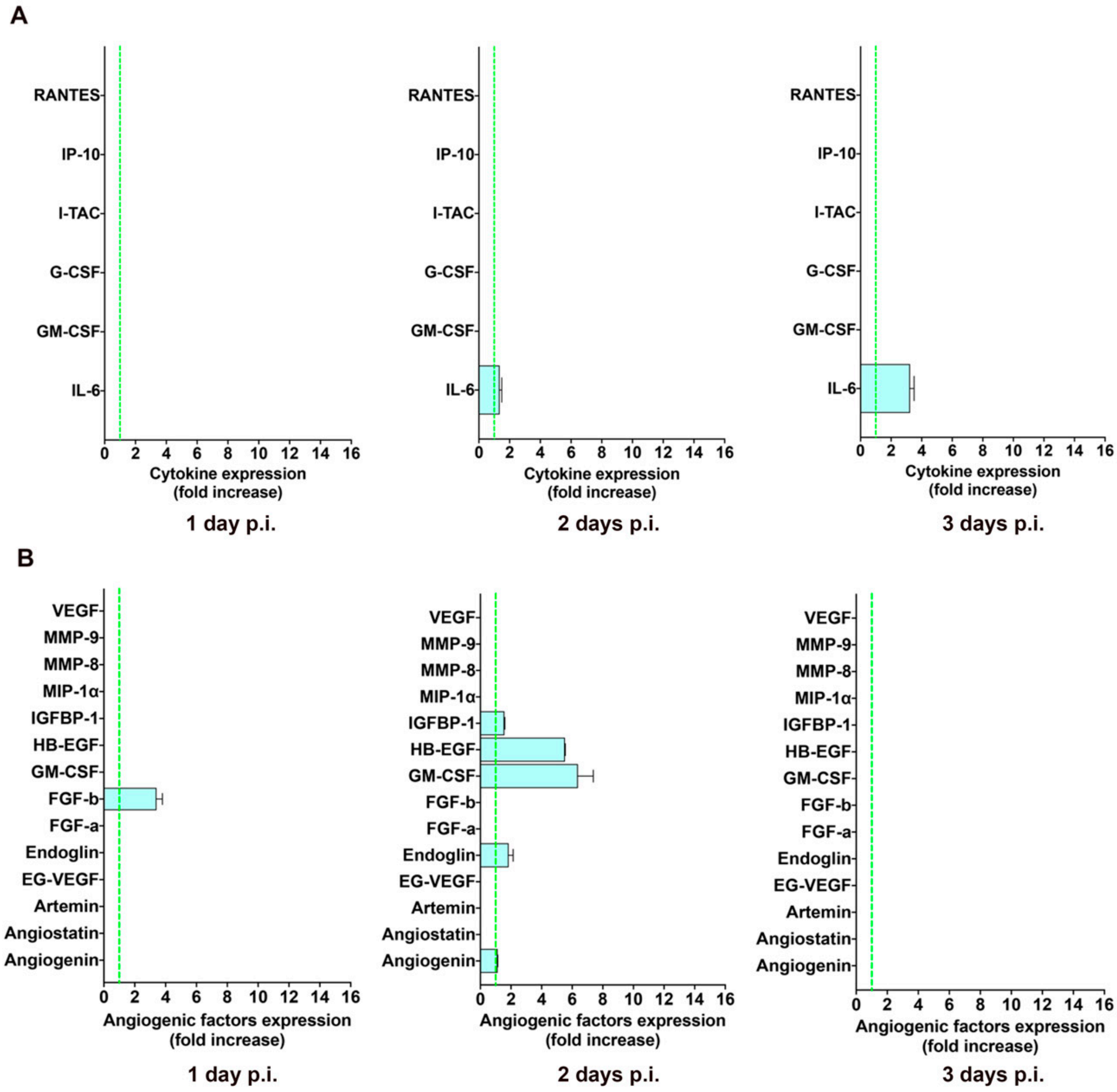

Figure S3. Release of cytokines and angiogenic molecules from mock-infected HL-mECs. HL-mECs were mock-infected (Mock) and cultured at 37°C for 3 days. Supernatants were evaluated at 1 (left panels), 2 (middle panels) and 3 (right panels) days of culture for the presence of (**A**) cytokines or (**B**) angiogenic molecules by human proteome arrays. The results are expressed as mean values ± SD of duplicates given as fold increase as compared to background (clear area of the array). Data are representative of one out of three independent experiments with similar results.

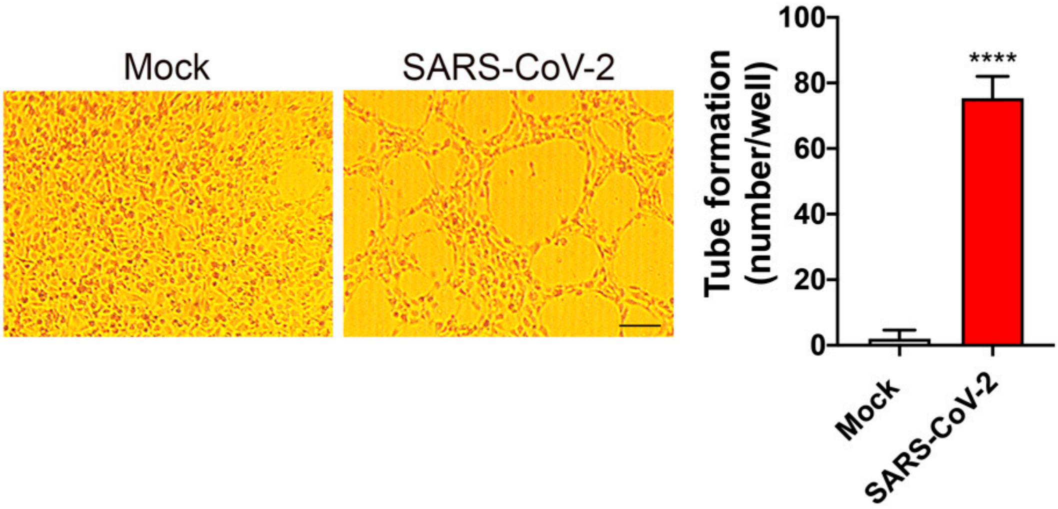

Figure S4. SARS-CoV-2 induces angiogenesis in HAECs. HAECs were mock-infected (Mock) or infected with SARS-CoV-2 (SARS-CoV-2) at a MOI 1, for 1 hour at 37°C and then washed and cultured until day 3 p.i.. Mock and SARS-CoV-2 HAECs were seeded on reduced growth factor Matrigel-coated wells and then cultured for 12 hours at 37°C. Pictures are representative of one out of three independent experiments with similar results. (Scale Bar, 200 µm). Values reported are the mean ± SD of one representative experiment out of three independent experiments with similar results performed in triplicate. Statistical analysis was performed by Student's two-tailed t-test (****P<0.0001).

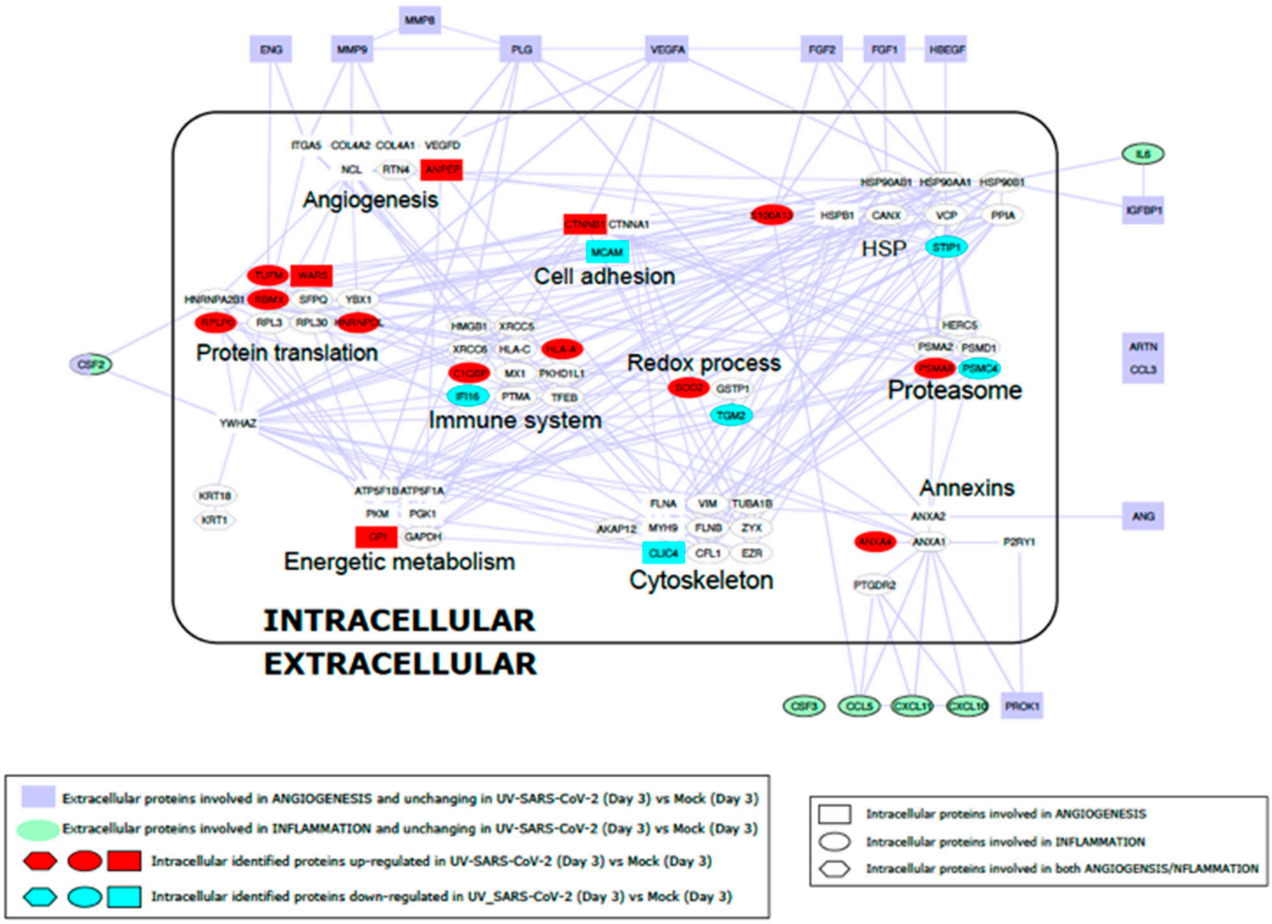

Figure S5. PPI network of DEPs involved in angiogenesis and inflammation at day 3 p.i. in UV-SARS-CoV-2 vs Mock-Infected HL-mECs. Interactome network (91 nodes and 262 edges) was built through the mapping of intracellular proteins found differentially expressed by proteomics and extracellular proteins assayed by angiogenic and inflammation array of UV-SARS-CoV-2 vs Mock HL-mECs. Physical or/and functional interactions connecting intra- and extra-cellular identified proteins are highlighted by thicker edges and considering experimental (STRING score > 0.15) and database (STRING score > 0.35) annotated interactions. The networks were visualized by Cytoscape 3.5 software, while biological processes were retrieved by BINGO Cytoscape's plugin. Intracellular node colour code indicates proteins up-regulated (in red) and down-regulated (in blue light) in UV-SARS-CoV-2- vs Mock-infected HL-mECs.

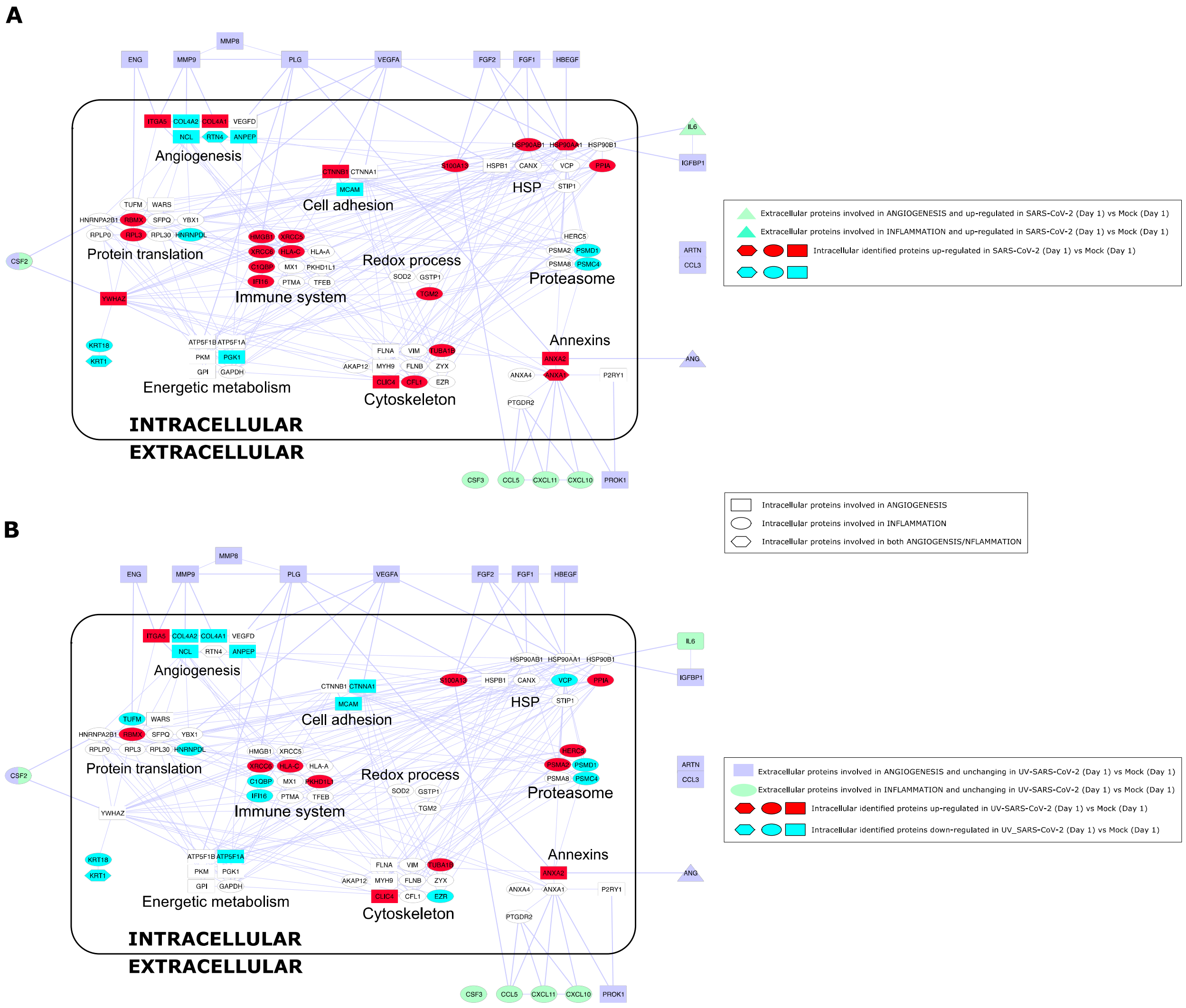

Figure S6. PPI network of DEPs involved in angiogenesis and inflammation at day 1 p.i.. Interactome network (91 nodes and 262 edges) was built through the mapping of intracellular proteins found differentially expressed by proteomics and extracellular proteins assayed by angiogenic and inflammation array of SARS-CoV-2 (**A**) and UV-SARS-CoV-2 (**B**) Conditions vs Mock at day 1 p.i. Physical or/and functional interactions connecting intra- and extra-cellular identified proteins are highlighted by thicker edges and considering experimental (STRING score > 0.15) and database (STRING score > 0.35) annotated interactions. The networks were visualized by Cytoscape 3.5 software, while biological processes were retrieved by BINGO Cytoscape's plugin. Intracellular node colour code indicates proteins up-regulated (in red) and down-regulated (in blue light) in SARS-CoV-2 (vs Mock) and UV-SARS-CoV-2 (vs Mock).

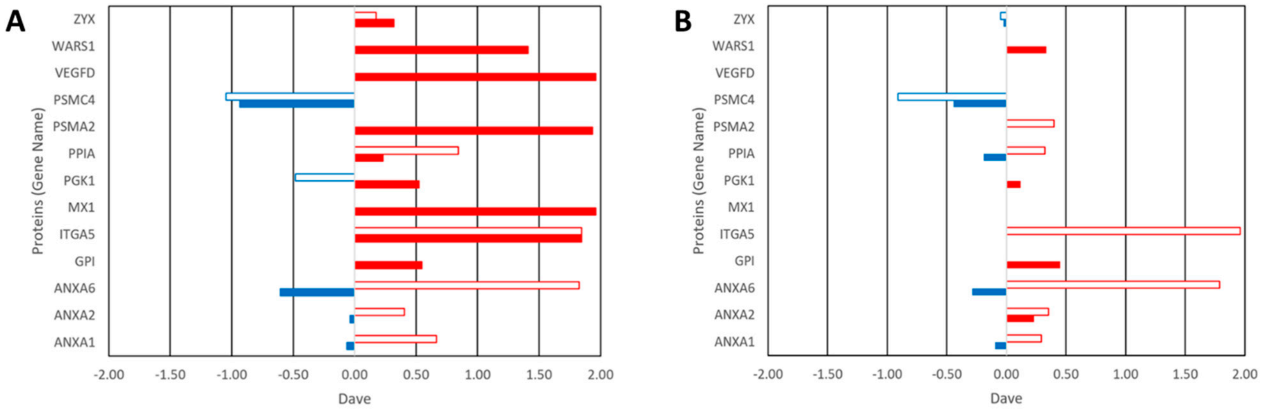

Figure S7. Differential analysis (by Dave) of the main extracted proteins. (**A**) SARS-CoV-2-infected HL-mECs and (**B**) UV-SARS-CoV-2-infected HL-mECs, compared to Mock-infected cells at day 1 (empty bars) and 3 (filled bars) p.i.. Red and blue bars correspond to the up- and down-expression of proteins vs Mock.

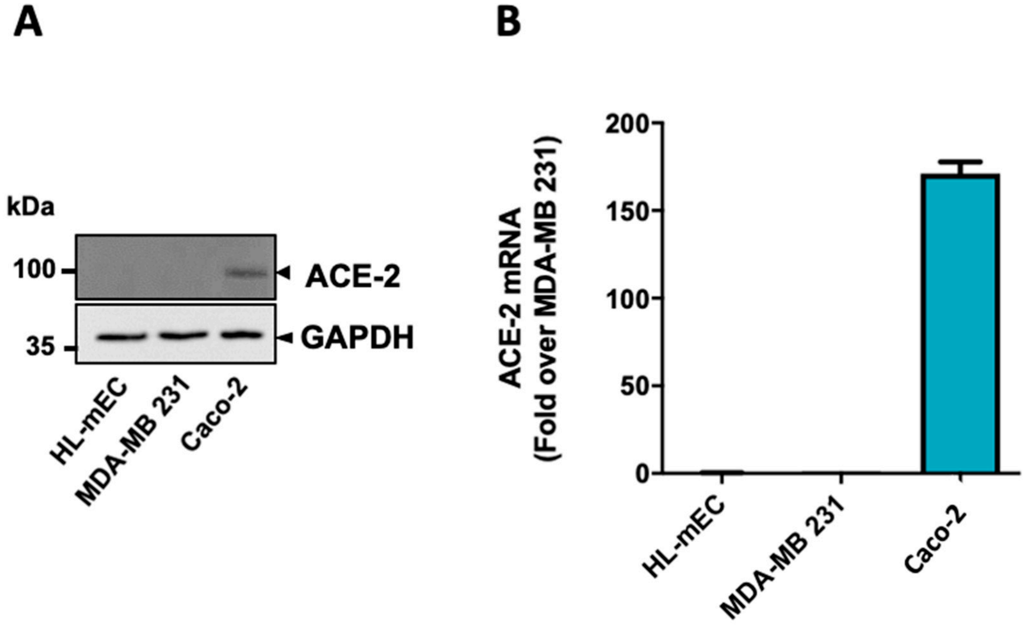

Figure S8. ACE2 expression in HL-mECs. (**A**) HL-mEC lysates were evaluated for expression of ACE2 by Western blot using mAb to ACE2 as a specific reagent. Blots from one representative experiment out of two with similar results are shown. Equal loading of cell extracts was evaluated by GAPDH protein staining, MDA-MB 231 and Caco-2 cell lines were used as a negative and positive control, respectively. (**B**) Analysis of ACE2 gene expression in HL-mECs performed by quantitative real-time PCR. MDA-MB 231 and Caco-2 cell lines were used as a negative and positive control, respectively.
